# Comparison of forced-air and water-circulating warming for prevention of hypothermia during transcatheter aortic valve replacement

**DOI:** 10.1371/journal.pone.0178600

**Published:** 2017-06-02

**Authors:** Benjamin Rohrer, Emily Penick, Farhad Zahedi, Hocine Tighiouart, Brian Kelly, Frederick Cobey, Stefan Ianchulev

**Affiliations:** 1 Department of Anesthesiology and Perioperative Medicine, Tufts Medical Center, Boston, MA, United States of America; 2 Tufts Clinical and Translational Science Institute, Boston, MA, United States of America; Harvard Medical School, UNITED STATES

## Abstract

**Introduction:**

Transcatheter Aortic Valve Replacement (TAVR) procedures at our institution were complicated by perioperative hypothermia despite use of the standard of care forced-air convective warming device (the BairHugger, Augustine Medical Inc, Eden Prairie, MN, USA). To remedy this problem, we initiated a quality improvement process that investigated the use of a conductive warm water-circulating device (the Allon ThermoWrap, Menen Medical Corporation, Trevose, PA, USA), and hypothesized that it would decrease the incidence of perioperative hypothermia.

**Methods:**

We compared two different intraoperative warming devices using a historic control. We retrospectively reviewed intraoperative records of 80 TAVRs between 6/2013 and 6/2015, 46 and 34 of which were done with the forced-air and water-circulating devices, respectively. Continuous temperature data obtained from pulmonary artery catheter, temperature upon arrival to cardiothoracic ICU (CTU), age, BSA, height, and BMI were compared.

**Results:**

Patients warmed with both devices were similar in terms of demographic characteristics. First recorded intraoperative temperature (mean 36.26 ± SD 0.61 vs. 35.95 ± 0.46°C, p = 0.02), lowest intraoperative temperature (36.01 ± 0.58 vs. 34.89 ± 0.76°C, p<0.001), temperature at the end of the procedure (36.47 ± 0.51 vs. 35.17 ± 0.75°C, p<0.001), and temperature upon arrival to the CTU (36.35 ± 0.44 vs. 35.07 ± 0.78°C, p<0.001) were significantly higher in the water-circulating group as compared to the forced-air group.

**Conclusion:**

A quality improvement process led to selection of a new warming device that virtually eliminated perioperative hypothermia at our institution. Patients warmed with the new device were significantly less likely to experience intraoperative hypothermia and were significantly more likely to be normothermic upon arrival to the CTU.

## Introduction

Since its introduction in the United States in 2011, the use of transcatheter aortic valve replacement (TAVR) has expanded dramatically, with the number of procedures reported in the Transcatheter Valve Therapy (TVT) Registry increasing from 4,590 to 26,414 between 2012 and 2014.**[1]** Trends in the anesthetic management of TAVR have changed as well. While general anesthesia remains by far the most common choice for TAVR in the US,**[2]** the use of moderate sedation is gradually increasing in popularity.**[1]** As the push continues toward faster operative times, more minimally invasive techniques, and shorter hospital stays, the prevention of hypothermia during TAVR becomes increasingly important as it predisposes to multiple perioperative complications,**[3–14]** is an important quality metric,**[3, 10, 15–17]** and is associated with increased costs.**[7, 9, 10, 14]**

Perioperative hypothermia has been defined by the Surgical Care Improvement Project as core temperature < 36.0°C.**[3, 11]** Hypothermia is thought to contribute to coagulopathy through slowing of enzyme reactions and impaired platelet adhesion and aggregation.**[8]** While core hypothermia has been well correlated with coagulopathy,**[8, 18]** even surface hypothermia in the setting of normal core temperature has been associated with altered coagulation parameters.**[19]** A seminal study by Frank *et al*. in patients with cardiac risk factors undergoing noncardiac surgery showed that even mild intraoperative hypothermia increased risk of ventricular tachycardia, unstable angina, ischemia, myocardial infarction, and cardiac arrest within the first 24 hours postoperatively. Furthermore, prevention of hypothermia in this study was associated with a 55% reduction in the risk for these events.**[6]** As such, prevention of hypothermia is likely to be of even greater relevance to TAVR patients known to be at prohibitive surgical risk.

A wide range of active rewarming devices are currently available.**[11]** These include electric resistive heating blankets, conductive warm water circulating systems, IV fluid warmers, infrared heating lights, negative pressure devices that aim to increase subcutaneous perfusion, and convective forced-air warming blankets. The latter have become the most widely adopted and best characterized in the literature.**[9, 10]** Initially developed as underbody mattresses, conductive warm water circulating systems more recently evolved into “garments” that can be wrapped around a patient’s body, increasing contact surface area.**[11]**

To improve perioperative temperature control at our institution, we undertook a quality improvement project using the Plan-Do-Study-Act (PDSA) methodology.**[20]** Hypothermia during TAVR at our institution was noted to be a common occurrence, with a high percentage of patients undergoing the procedure between June 2013 and April 2014 dropping below 36.0 degrees Celsius at some point despite use of an underbody forced air warming device. The reasons for this were thought to be multifactorial, and included large exposed surface, concerns over the detrimental impact of increased ambient temperatures on measures of surgical performance,**[21]** and the use of a forced-air warming device that could not be activated until after draping had been completed due to theoretical concerns about increased rates of wound infection.**[11, 22, 23]** We addressed this problem by evaluating all aspects of patient warming, including ease of use and acceptability to all staff involved in the procedure. We selected a device which conformed better to the entire process and allowed for earlier initiation of warming.**[24]** We hypothesized that this new conductive warming device would decrease the incidence of periprocedural hypothermia, with the ultimate goal of having all patients normothermic at the end of the procedure.

## Materials and methods

Approval was granted by the Tufts Medical Center Internal Review Board. No specific written consent was obtained for this retrospective observational quality improvement study as all patient data were anonymized prior to analysis.

### Data collection

We retrospectively reviewed intraoperative records from 80 TAVR procedures performed under general anesthesia between June 2013 and June 2015, 46 and 34 of which were done with the forced-air and water-circulating devices, respectively. Hypothermia was defined as temperature below 36.0°C. This threshold was adopted from the Surgical Care Improvement Project (SCIP) guideline Inf-10, compliance with which has been associated with improved clinical outcomes.**[3]** We evaluated our results with the goal of having our patients at or above 36.0° Celsius upon arrival to the cardiothoracic ICU (CTU).

Continuous temperature data obtained from pulmonary artery catheter, temperature upon arrival to CTU, age, BSA, height, weight, surgical approach (transfemoral versus transapical), surgeon, and length of surgery were compared. IV fluids, blood products, surgical time, and anesthesia time were obtained from intraoperative records. Intraoperatively, all fluids were given through a fluid warming device set to 41°C.

### Anesthetic methods

General anesthesia was used in all cases, with an arterial line placed preoperatively for hemodynamic monitoring. A central line and pulmonary artery catheter were placed post-induction. Active warming was initiated after prepping and draping in the forced-air warming group, and immediately upon moving to the OR table in the water-circulating group. The forced-air and water-circulating warmers were set to 43 and 37°C, respectively. The forced-air warming device was in contact with the entire posterior surface of patients’ bodies except for the back of the head. The water-circulating device was in contact with the posterior surface of patients’ bodies from the trunk down to the thighs and wrapped around both upper extremities. Each device covered approximately 45% of total body surface area. Both warming devices were used according to the manufacturer’s instructions. Induction agents, pressors, blood products, and IV fluids were administered according to provider judgement rather than a set protocol. During the study period, all patients were extubated in the CTU rather than in the OR.

### Statistical analysis

Mean temperature over time was estimated with a random effect mixed model using B splines for time and including an interaction between time and warming device. Additionally, to quantify duration and degree of hypothermia in both groups, we used continuous temperature recordings to derive an “area under threshold” measure, defined as size of the region above the temperature versus time curve but below 36.0 degrees Celsius. We used the t-test and Wilcoxon rank sum tests for continuous variables as appropriate, and Pearson Chi-square test for categorical variables to compare differences between groups. An a priori p value of 0.05 was used to delineate statistical significance. A mixed statistical model was also used to control for the effects of BSA, anesthesia duration, surgical approach, and crystalloid volume on temperature in both groups.

## Results

There were no statistically significant differences between the forced-air and water-circulating groups for age (mean 80 ± SD 9 vs. 80 ± 9 years, p = 0.96), body surface area (1.9 ± 0.3 vs. 1.8 ± 0.2 m^2^, p = 0.18), and height (167 ± 9 vs. 165 ± 10 cm, p = 0.49). There was a non-significant trend towards transfemoral approach being more common in the water-circulating group compared to the forced-air group (16 (47%) vs. 15 (33%), p = 0.19). There was also a non-significant trend towards patients in the forced-air group having higher BMI compared to those in the water-circulating group (28.6 ± 7.5 vs, 26.3 ± 4.0 kg/m^2^, p = 0.09). Procedure time was significantly shorter in the water-circulating group as compared to the forced-air group (134 ± 48 vs. 162 ± 42 minutes, p = 0.009) (Table 1).

**Table 1:** Demographic characteristics and intraoperative variables.

		Forced-air	Water-circulating	P value
Age (year, mean ∓ SD)		80.5 ± 8.7	80.4 ± 8.8	0.96
Height (cm, mean ∓ SD)		166 ± 9	165 ± 10	0.49
BMI (kg/m^2^, mean ∓ SD)		28.6 ± 7.5	26.3 ± 3 4	0.09
BSA (m^2^, mean ∓ SD)		1.9 ± 0.3	1.8 ± 0.2	0.18
Surgical approach	Transfemoral (n)	15	16	0.19
	Transapical (n)	31	18	
Anesthesia time (minutes, mean ∓ SD)		258 ± 43	235 ± 49	0.03
Procedure time (minutes, mean ∓ SD)		162 ± 42	134 ± 48	0.009
Crystalloid (mL, mean ∓ SD)		1687 ± 639	1391 ± 569	0.04
PRBC (units, mean ∓ SD)		0 ± 0	1 ± 1	0.12
FFP (units, mean ∓ SD)		0 ± 0	0 ± 1	0.10
Cryoprecipitate (units, mean ∓ SD)		0 ± 0	0 ± 0	0.32
Cell saver (mL, mean ∓ SD)		43 ± 122	94 ± 327	0.39
Platelets (units, mean ∓ SD)		0 ± 0	0 ± 1	0.10
Blood loss (mL, mean ∓ SD)		50 ± 172	103 ± 175	0.18

**Fig 1:**
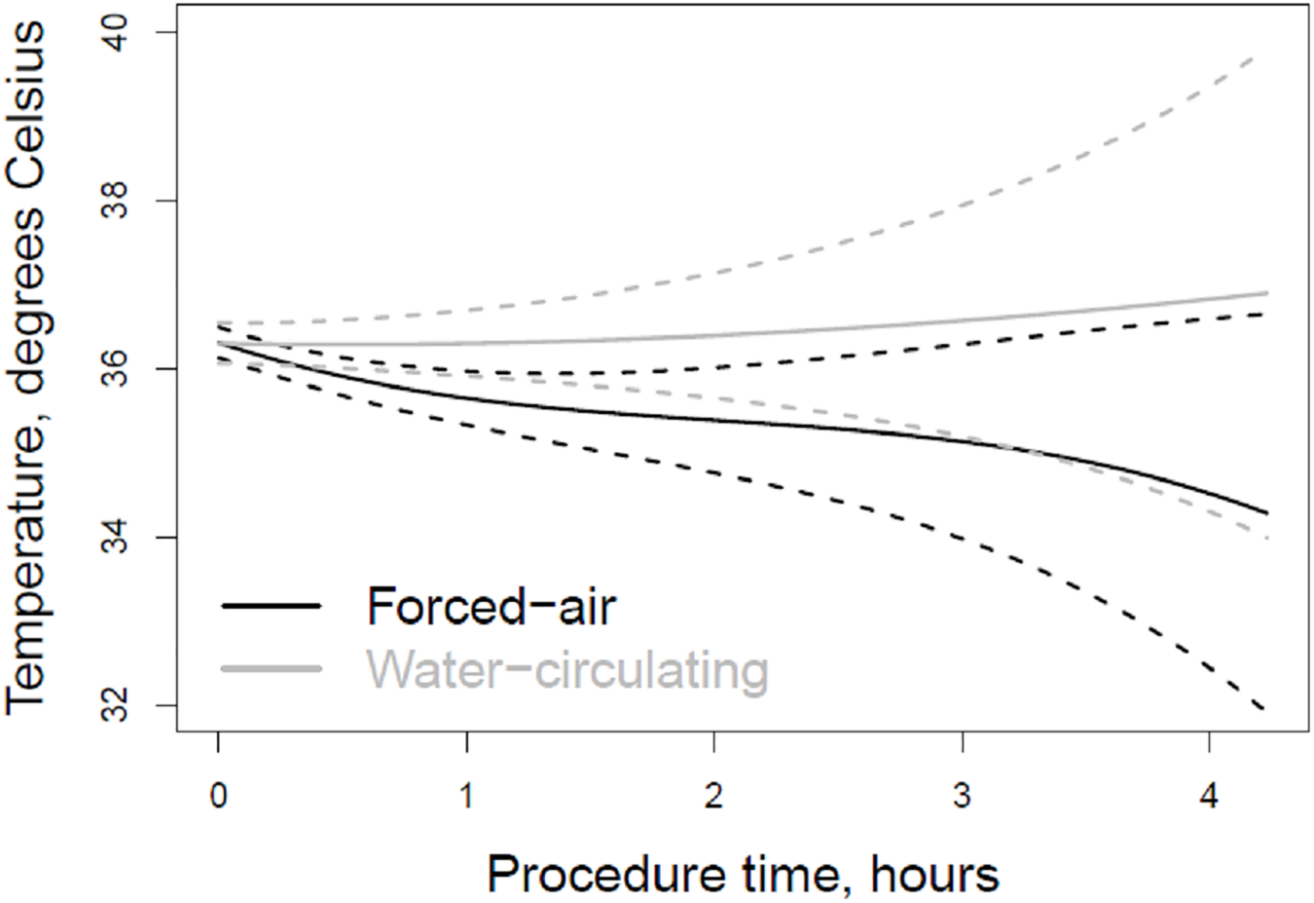
Mean temperature versus time in TAVR patients warmed with forced-air and water-circulating devices. Temperature decreased significantly over time in the forced-air group (P < 0.001), and the trajectories for the two groups were significantly different (p = 0.004). Dashed lines represent 95% confidence interval. Mean temperature over time was estimated with a random effect mixed model using B splines for time, and included an interaction between time and device status. The number of patients present at the 1, 2, 3, and 4-hour time points in the forced-air and water-circulating groups were 46 and 34, 43 and 19, 12 and 3, and 1 and 1, respectively.

**Fig 2:**
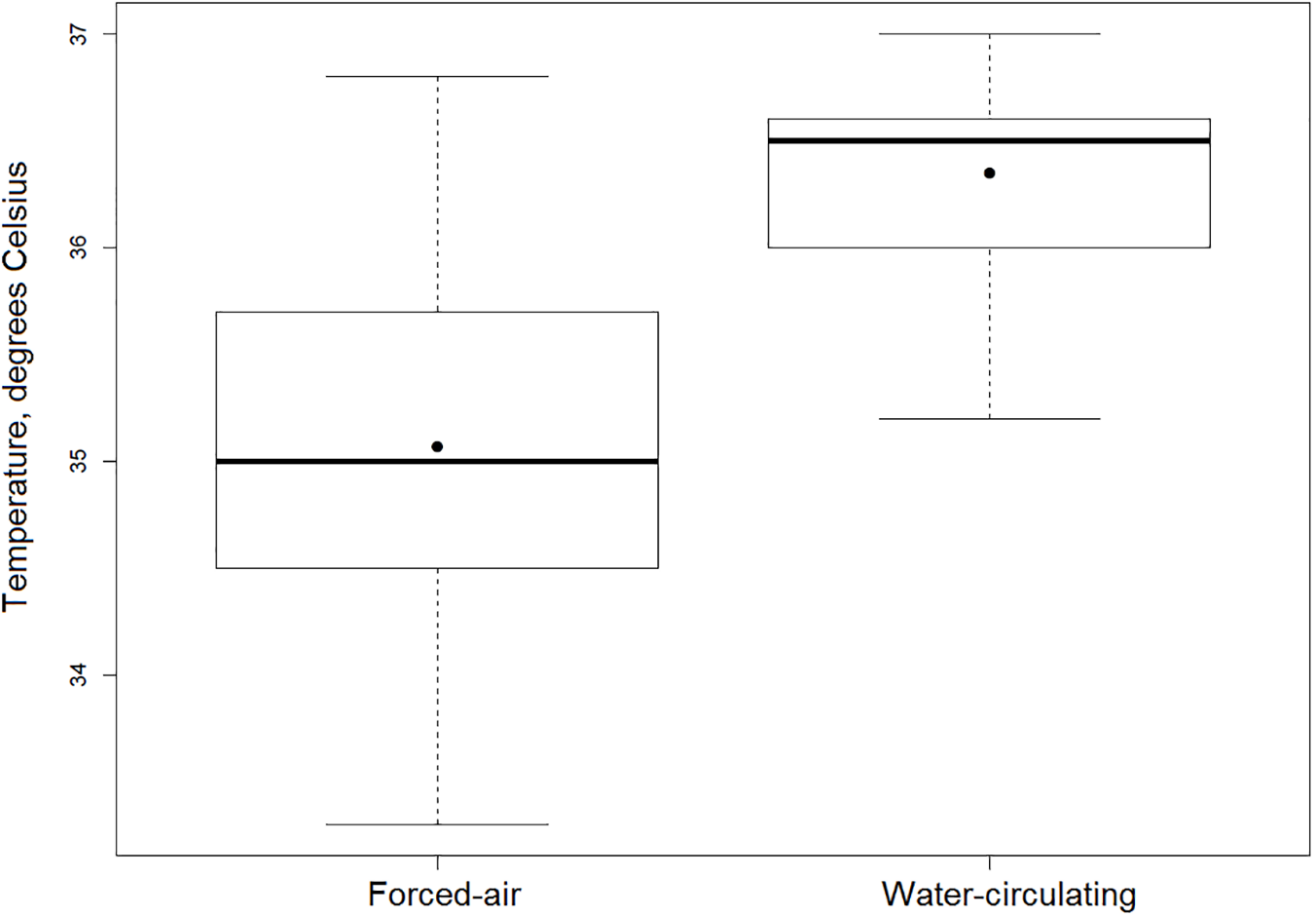
Temperature on arrival to the CTU. Mean temperatures ± SD for patients warmed with the forced-air and water-circulating devices were 36.4 ± 0.4 and 35.1 ± 0.8 degrees Celsius, respectively (p < 0.001). Overall, 97% of patients warmed with the water-circulating device versus 13% of those warmed with the forced-air device were above 36.0 degrees Celsius upon arrival to the CTU (p < 0.001).

**Fig 3:**
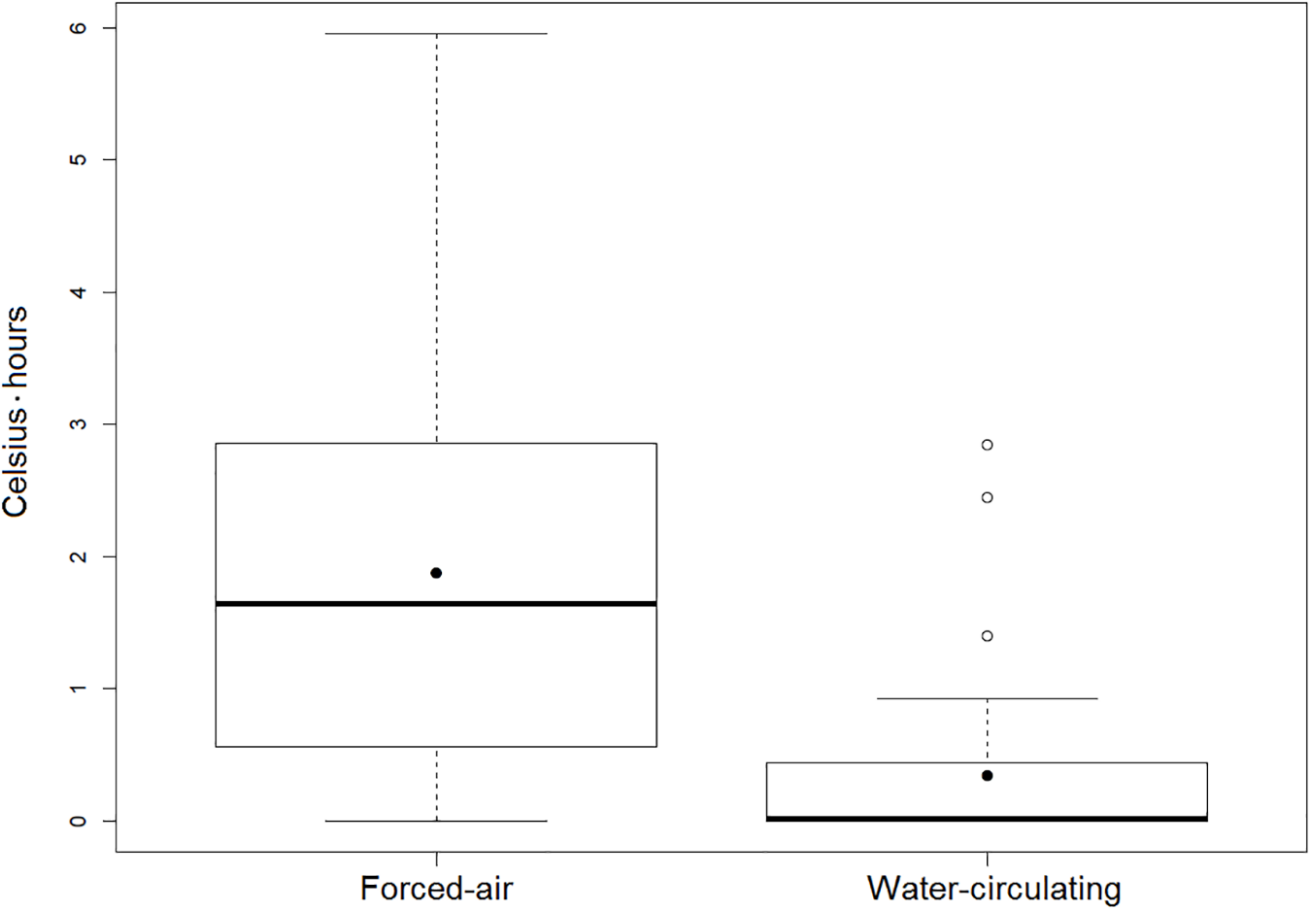
Degree-hours below 36 degrees Celsius. Area between mean temperature versus time curve and below 36.0 degrees Celsius for patients warmed with forced-air and water-circulating devices. The median areas under 36.0 degrees Celsius were 0.0 and 1.6 degree-hours for the forced-air and water-circulating groups, respectively. The duration and magnitude of hypothermia was significantly greater in the forced-air warming group (p < 0.001).

First recorded intraoperative temperature (36.2 ± 0.6 vs. 36.0 ± 0.5°C, p = 0.02), lowest intraoperative temperature (36.0 ± 0.6 vs. 34.9 ± 0.8, p<0.001), temperature at the end of the procedure (36.5 ± 0.5 vs. 35.2 ± 0.8°C, p<0.001), and temperature upon arrival to the ICU (36.4 ± 0.4 vs. 35.1 ± 0.8°C, p<0.001) were significantly higher in the water-circulating group as compared to the forced-air group. This effect remained significant even after applying a mixed model to account for BSA, anesthesia duration, surgical approach, and crystalloid volume (p<0.001). Mean temperature decreased significantly over time in the forced-air group (p<0.0001) (Fig 1). In the water-circulating group there were 33 (97%) patients with temperature greater than 36 degrees Celsius upon arrival to the ICU compared to 6 (13%) in the forced-air group (p<0.001) (Fig 2). Additionally, degree-hours under 36 degrees Celsius was also significantly lower in the water-circulating group compared to the forced-air group, median (25^th^, 75^th^ percentiles) 0.0 (0.0, 0.4) vs. 1.6 (0.6, 2.9) Celsius*hours, (p<0.001) (Fig 3).

## Discussion

Our results supported the hypothesis that the use of water-circulating conductive heating would reduce the incidence of periprocedural hypothermia in TAVR as compared to the use of convective forced-air warming. While a 2016 meta-analysis identified nine studies comparing forced-air and water-circulating devices for the prevention of intraoperative hypothermia, only four examined patients undergoing cardiothoracic procedures.**[25–28]** Of these, all were restricted to patients undergoing off-pump coronary artery bypass grafting. In contrast, our study looked specifically at the TAVR population.

We observed a significant difference in procedural time between the two groups, with procedures lasting approximately 30 minutes longer on average in the forced-air warming group. This is likely a reflection of this group being a historical control, with operator experience improving over time. This difference in surgical times by itself is unlikely to explain the observed differences in perioperative temperatures, as maximum redistribution heat loss is usually reached around the one-hour mark. Rather than leading to further heat loss, additional procedure time beyond one hour actually affords an opportunity for rewarming.**[10]** Interestingly, we found that patients in the forced-air warming group actually tended to get colder over time (Fig 1).

We also observed non-significant trends towards more frequent use of the transapical approach and higher BMI in the forced-air group, as well as higher blood loss and blood product usage in the water-circulating group. Differences in procedural approach were unlikely to have influenced temperature data, since all patients were prepared and draped in an identical manner to allow for use of the transapical route should problems arise with transfemoral access. Additionally, surgical approach did not have a significant impact on the relationship between warming device and temperature upon arrival to the CTU in our mixed statistical model. While there was a trend towards higher BMI in the forced-air group, this is unlikely to have contributed to hypothermia since increased adipose tissue is actually associated with retention of heat during anesthesia.**[29]** Similarly, while there was a trend towards higher blood loss in the conductive warming group, the finding that these patients were nevertheless warmer during and after surgery further supports our hypothesis (Table 1).

Although there is abundant literature on the subject of intraoperative hypothermia, studies comparing the intraoperative use of convective forced-air and conductive water-circulating warmers for the prevention of perioperative hypothermia are relatively scarce. In their meta-analysis of nine studies looking at the effectiveness of intraoperative warming for prevention of perioperative complications, Madrid *et al*. found no evidence of superiority of one device over another in terms of clinical outcomes. However, they did report non-significant statistical trends towards lower rates of surgical site infection, myocardial infarction, and all-cause mortality in patients warmed with a conductive water-circulating device.**[9]** With further investigation the relationship between these outcomes and choice of intraoperative warming device might be further borne out.

### Limitations

Limitations to our study include its use of a historical control, and its single-center, retrospective design. Our use of a historical control may have influenced our results, as TAVR is a complex procedure that requires coordination across multiple disciplines, and it is likely that procedure time decreased as our staff grew more experienced. Our conclusions are also limited by the pre-induction initiation of warming only in the water-circulating group, and by the trend towards more frequent usage of transapical access in the forced-air group.

## Conclusion

A quality improvement process led to selection of a new warming device that virtually eliminated perioperative hypothermia at our institution. Patients warmed with the new device were significantly less likely to experience intraoperative hypothermia and were significantly more likely to be normothermic upon arrival to the CTU. Based on the results of this study, we have expanded our use of the water-circulating warming device to all cardiac surgical patients as the next step in our quality improvement initiative. While the clinical implications of improved temperature management were beyond the scope of this study, we hope to examine this topic in future investigations.
